# Efficacy and safety of the traditional Chinese medicine formulation BL-1 for the treatment of low anterior resection syndrome: study protocol for a double-blind, randomized, placebo-controlled trial

**DOI:** 10.3389/fgstr.2026.1767318

**Published:** 2026-07-30

**Authors:** Jun Zhao, Jianxia Dong, Yu Zhang, Shi-Yu Tang, Qing Li, Qiangqi Cai, Xuemei Zhang, Lie Yang, Lin Zhu

**Affiliations:** 1 Department of Integrated Traditional and Western Medicine, West China Hospital, Sichuan University, Chengdu, China; 2 Department of Pharmacy, West China Hospital, Sichuan University, Chengdu, China; 3 Department of Gastrointestinal Surgery, West China Hospital, Sichuan University, Chengdu, China; 4 School of Pharmacy, Chengdu Medical College, Chengdu, China

**Keywords:** clinical trial, LARS, postoperative treatment, protocol, traditional Chinese medicine

## Abstract

**Objective:**

Low anterior resection syndrome (LARS), a prevalent sequela following rectal cancer surgery, poses a substantial burden on patients’ quality of life (QOL), daily functioning, and psychological well-being, and its management remains an unmet clinical need due to the absence of specific therapeutic agents. Although traditional Chinese medicine (TCM) shows promise in supporting postoperative gastrointestinal recovery, high-quality evidence for its application in LARS is lacking. This trial is designed to assess the efficacy and safety of the investigational TCM formulation BL-1 in patients with LARS.

**Methods/design:**

The study will be conducted as a randomized, double-blind, placebo-controlled trial, comprising a 4-week intervention phase and a subsequent 2-week follow-up period. A total of 234 patients with LARS, aged 18 to 75 years, are planned to be enrolled in this study. Eligible participants will be randomized to receive either the BL-1 group or placebo group in a 1:1 ratio. Participants in the experimental group will receive BL-1 formulation, whereas those in the placebo group will receive an indistinguishable BL-1 placebo. The primary outcome measure is the change in LARS score from baseline to week 4. Secondary outcome measures encompass: (a) stool frequency and consistency (assessed via the Bristol Stool Scale); (b) severity of fecal incontinence (evaluated using the Wexner Anal Incontinence Scale); (c) QOL, measured with the European Organization for Research and Treatment of Cancer QOL questionnaire and the Karnofsky Performance Status score; and (d) other patient-reported outcomes. Safety will be monitored continuously throughout the treatment, with assessments including the incidence of adverse events and laboratory abnormalities.

**Discussion:**

BL-1 is the compound prescription of eleven traditional Chinese medicinal materials: Bupleuri Herba, Ginseng Radix et Rhizoma, Astragali Radix, Atractylodis Macrocephalae Rhizoma, Cimicifugae Rhizoma, Mume Fructus, and Aconiti Lateralis Radix Praeparata (patent number ZL 2024 1 1479878.8). In recent years, BL-1 has been applied to treat digestive system diseases, particularly defecation disorders, and may offer potential benefits for adults with LARS. The absence of robust clinical evidence has prompted the design of this randomized, double-blind, placebo-controlled trial to rigorously evaluate its therapeutic efficacy and safety. This trial is expected to yield critical data that will inform the clinical use of BL-1 formulation in the management of LARS.

**Ethics and dissemination:**

Ethical approval for this study was obtained from the Ethics Committee of West China Hospital, Sichuan University (Approval ID: 20241218). All participants must provide written informed consent before randomization. Subsequently, the trial results will be submitted for publication in a peer-reviewed academic journal.

**Clinical trial registration:**

https://www.chictr.org.cn, identifier ChiCTR2400087108.

## 1 Introduction

Colorectal cancer is the third most commonly diagnosed cancer globally, with nearly 50% of these cases originating in the rectum (1). According to the statistics of the China Cancer Centre, the fatality rate of colorectal cancer ranks fourth, and the incidence rate continues to increase (2). Current management of rectal cancer primarily relies on surgery, with sphincter-preserving techniques being highly prioritized provided that oncological outcomes are not compromised. Low anterior resection and/or total mesorectal excision are the preferred surgical methods for radical resection of low rectal cancer because the can avoid the need for a lifelong stoma (3, 4). This intervention, however, induces significant alterations in anorectal physiology, giving rise to a spectrum of defecatory abnormalities collectively referred to as low anterior resection syndrome (LARS). With an incidence ranging from 25% to 80%, this syndrome manifests predominantly as fecal incontinence, defecatory urgency, and elevated bowel movement frequency (5). While some symptomatic improvement may occur between 6 months and 1 year postoperatively (6, 7), a majority of patients continue to experience long-term disordered bowel function after rectal resection. These persistent alterations commonly include unpredictable bowel habits, changes in stool consistency, increased frequency, painful defecation, emptying difficulties, urgency, incontinence, and soiling (8). These defecatory problems represent a primary source of patient distress post-surgery and substantially compromise overall quality of life (QOL) (9, 10).

The etiology and pathogenesis of LARS are multifactorial, primarily involving three interrelated mechanisms. (1) the neorectum’s impaired reservoir function leads to reduced storage capacity. (2) direct injury to the anal sphincter complex or its innervation during surgical dissection or anastomosis is a critical determinant. (3) preoperative radiotherapy and postoperative complications (e.g., anastomotic leakage) further contribute to the symptomatic spectrum (11). The underlying pathogenesis of LARS encompasses several physiological disturbances, such as internal anal sphincter dysfunction, diminished rectal sensitivity and compliance, and the loss of the rectoanal inhibitory reflex (11). Disruption of the pelvic neural plexus during low anterior resection may result in aberrant reinnervation of the neorectum, a process that can contribute to the exacerbation of LARS symptoms (12). However, current therapies, including diet and lifestyle modifications, pelvic floor muscle training, transanal irrigation, antidiarrheal drugs, 5-hydroxytryptamine (5-HT) receptor antagonists, fiber, probiotics and even neuromodulation, are not always effective (9, 11). The high incidence of rectal cancer in our country underscores the urgency for developing safe and effective pharmacological interventions for LARS, which would hold substantial clinical and societal value.

While LARS is not a defined disease entity in Traditional Chinese Medicine (TCM), its clinical manifestations can be understood and categorized within TCM theory primarily as patterns of diarrhea and constipation, based on the predominant symptoms. Modern surgical removal of cancerous tumors falls under the category of ‘eliminating pathogens’ (addressing symptoms), but the pathological mechanism of LARS, ‘spleen qi deficiency’ (the root cause), persists throughout patients at different stages and severity levels. (1) Weakness of the spleen and stomach leads to sinking of clear yang and inadequate lifting, resulting in rectal prolapse and incontinence; (2) deficiency of yang energy in the large intestine results in dysfunctional fecal discharge, primarily presenting as incomplete defecation. TCM offers a comprehensive therapeutic system for managing LARS, which includes internal and external methods. The TCM management strategy incorporates both external methods (such as electroacupuncture, external application, and moxibustion) and internal approaches (primarily involving oral traditional Chinese herbal formulas and oral Chinese patent medicines). Previous studies have shown that the Chinese herbal formulas Buzhong YiQi (13, 14) and Shenling Baizhu San (15) can improve LARS symptoms in patients after low anterior resection of rectal cancer in the short term. However, studies published to date have small sample sizes, and a high proportion of clinical trials were conducted with nonrandomized designs in, which limits the generalizability of the clinical findings of these studies for the above Chinese herbal compound formulas.

BL-1 is a proprietary herbal formulation developed by West China Hospital of Sichuan University. Our team’s preliminary small-sample pilot study results indicate that BL-1 has an efficacy rate of approximately 65% in treating LARS (data not yet published). It contains various key components, including but not limited to Bupleuri Herba, Ginseng Radix et Rhizoma, Astragali Radix, Atractylodis Macrocephalae Rhizoma, Cimicifugae Rhizoma, Mume Fructus, and Aconiti Lateralis Radix Praeparata (patent number ZL 2024 1 1479878.8). BL-1 aims to treat the root-cause of LARS with “sun-uplift and dispel cold and dampness” and its symptoms with “astringent intestines and stop”. This formulation is particularly suitable for LARS patients diagnosed with “Yangming deficiency cold syndrome”, especially those with urgent fecal incontinence. Consequently, a randomized, multiple-blind, placebo-controlled trial is designed to assess the efficacy and safety of BL-1 formulation for treating adults with fecal incontinence-type LARS.

## 2 Methods/design

### 2.1 Study design

**Figure 1:**
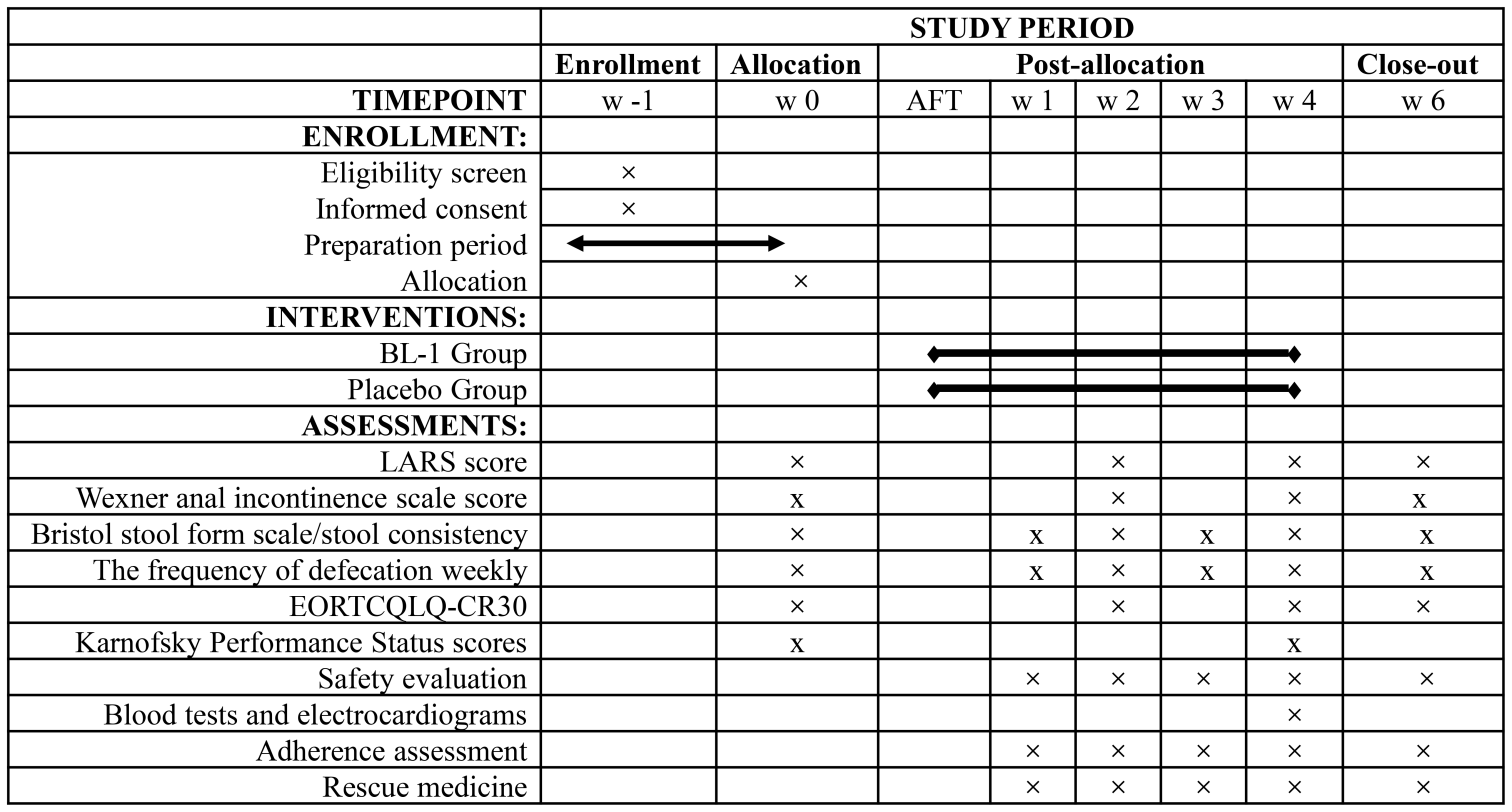
SPIRIT schedule. Blood tests or verify results are required during the Preparation period BL-1, a proprietary herbal formulation developed by West China Hospital of Sichuan University; LARS, low anterior resection syndrome; EORTC QLC-CR30, the scale of cancer-specific European Organization for Research and Treatment of Cancer; AFT, prior to the initiation of therapy.

This study is a multicenter, randomized, double-blind, placebo-controlled exploratory trial. The protocol was developed in accordance with the Standard Protocol Items: Recommendations for Interventional Trials guidelines (16). **Figure 1** presents the trial timeline and assessment schedule. Eligible participants will be randomized to receive either the BL-1 formulation or placebo at a 1:1 ratio. The trial for both groups will comprise a 4-week intervention period followed by a 2-week follow-up. Ethical approval was obtained from the Ethics Committee of West China Hospital, Sichuan University (Approval ID: 20241218). The trial was prospectively registered in the Chinese Clinical Trial Registry under the identifier ChiCTR2400087108.

### 2.2 Trial status

The protocol has been updated to version V4.1.1 (revised October 2025). To date, 92 participants have been enrolled, with completion anticipated by June 2027. The study results are expected to be published in December 2027.

### 2.3 Trial setting

We will recruit 234 LARS patients from the outpatient clinic or inpatient ward of hospitals in Chengdu, China: West China Hospital of Sichuan University, Sichuan Cancer Hospital, and the Affiliated Hospital of Chengdu University of Traditional Chinese Medicine.

### 2.4 Participants

#### 2.4.1 Recruitment

Recruitment strategies will include hospital social media (e.g., WeChat subscriptions), digital posters (e.g., WeChat Moments), on-site postings in outpatient clinics and wards, as well as advertisements in surrounding community service centers: 1) the recruitment posters (both print and electronic) will outline the key eligibility criteria for potential participants and provide details regarding the trial activities and commitments involved; 2) in clinical settings (outpatient and inpatient), physicians will initially identify potential candidates suspected of having LARS for a subsequent detailed evaluation to determine final eligibility. Simultaneously, we will provide contact information for participants as well as gastroenterologists and gastrointestinal surgeons. The Clinical Research Coordinator will evaluate the eligibility of potential participants through post-surgical interviews, applying the study’s inclusion and exclusion criteria. All included patients or their legally authorized representatives will voluntarily agree to participate and will sign an informed consent document before randomization; 3) participant adherence will be enhanced through a strategy of regular telephone follow-ups.

#### 2.4.2 Participant inclusion criteria

The key inclusion criteria are as follows: (a) aged 18–75 years; (b) a preoperative clinical diagnosis of rectal cancer with a TNM stage I–III; (c) patients meeting the 2020 LARS diagnostic criteria with a LARS score ≥ 20; (d) transanal defecation frequency ≥ 4 times/day; (e) rectal cancer anal sphincter preservation surgery (including preventive stoma retraction surgery) performed at least one month prior; (f) a Karnofsky score > 60 and an expected survival > 6 months; (g) patients with basic communication skills and understanding who are willing to cooperate with the examination, treatment and follow-up; and (h) patients who have provided signed informed consent following a detailed explanation of the trial.

#### 2.4.3 Participant exclusion criteria

The exclusion criteria are as follows: (a) patients undergoing postoperative radiotherapy and chemotherapy; (b) patients with an established etiology for diarrhea other than LARS (e.g., Crohn’s disease, irritable bowel syndrome, or other known causes); (c) patients with local tumor recurrence; (d) patients with the presence of serious postoperative complications, exemplified by anastomotic leakage or stenosis; (e) patients with concomitant liver dysfunction (alanine aminotransferase or aspartate aminotransferase exceeding 1.5 times the upper limit of normal) and renal dysfunction (chronic kidney disease stage 4 or above); (f) patients with severe brain, heart, and other systemic diseases; (g) individuals with neurological or mental disorders who are unable or unwilling to cooperate; (h) patients with legally defined disabilities (e.g., visual/hearing/speech impairment or intellectual disability); (i) individuals suspected or confirmed to have a recent history of alcohol abuse, drug misuse, or illicit substance use; (j) pregnant or lactating women or individuals with a near-term pregnancy plan; and (k) those who have been treated with TCM during the past 3 months or who have participated in other clinical trials.

#### 2.4.4 Trial withdrawal

Patients who require reoperation or chemoradiotherapy due to disease progression, as well as those who withdraw from the study after randomization for any other reason and subsequently cease participation in the intervention or follow−up, will be considered withdrawn from the study. Upon dropout, the investigator is expected to make all reasonable attempts to reach the individual, document the cause of withdrawal, and complete relevant assessments. All data related to the dropout cases should be preserved appropriately for documentation.

### 2.5 Intervention

#### 2.5.1 The experimental group

Participants will be provided with the BL-1 formulation by the West China Clinical Pharmacy Department (Chengdu, China). Some details on BL-1 are listed in **Table 1**. This compound formulation is a water extract of various traditional Chinese medicinal materials: Bupleuri Herba 15g, Ginseng Radix et Rhizoma 15g, Astragali Radix 30g, Atractylodis Macrocephalae Rhizoma 15g, Cimicifugae Rhizoma 15g, Mume Fructus 15g, and Aconiti Lateralis Radix Praeparata 15g (patent number ZL 2024 1 1479878.8). The formulation is supplied at 100ml/bag. Oral supplements were taken three times daily before meals for 4 weeks. The expected adverse effects of BL−1 include nausea, abdominal pain, bloating, constipation, and others.

**Table 1:** Components of BL-1 (patent number ZL 2024 1 1479878.8).

Latin/scientific name	Chinese pinyin	Dose	Sources	Parts or form used
Bupleuri Herba	Chaihu	15g	*Bupleurum marginatum* Wall. ex DC.	Dried
Ginseng Radix et Rhizoma	Rensheng	15g	*Panax ginseng* C. A. Mey.	Dried root
Astragali Radix	Huangqi	30g	*Astragalus membranaceus* (Fisch.) Bge. *var. mongholicus* (Bge.) Hsiao	Dried root
Atractylodis Macrocephalae Rhizoma	Baizhu	15g	*Atractylodes macrocephala* Koidz.	Dried root
Cimicifugae Rhizoma	Shengma	15g	*Cimicifuga foetida* L.	Dried root
Mume Fructus	Wumei	15g	*Prunus mume* (Sieb.) Sieb. et Zucc.	Dried unripe fruit
Aconiti Lateralis Radix Praeparata	Fuzi	15g	*Aconitum carmichaelii* Debx.	Dried root

#### 2.5.2 The control group

**Figure 2:**
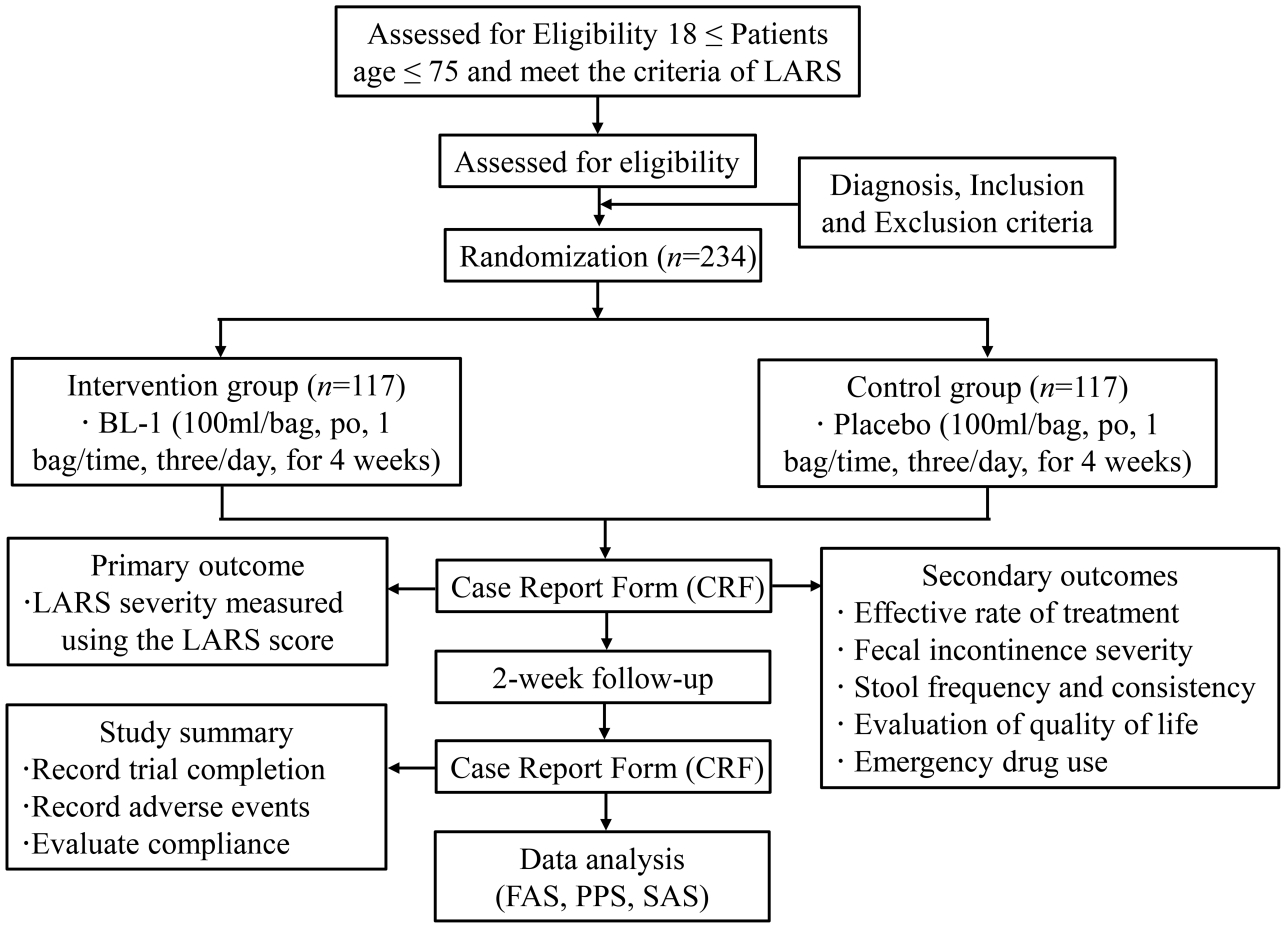
Flow chart of the study design. BL-1, a proprietary herbal formulation developed by West China Hospital of Sichuan University; LARS, low anterior resection syndrome; FAS, full analysis set; PPS, per-protocol set; SAS, safety set.

The placebo, manufactured by the West China Clinical Pharmacy Department (Chengdu, China), will be orally administered at a dose of one bag three times daily before meals over a 4-week course. The placebo is a low-dose mixture prepared using 10% of the therapeutic group’s prescribed dosage. Its appearance and specifications are designed to be indistinguishable from those of the BL-1 formulation. Additionally, post−hoc testing will be performed to verify the validity of blinding. A flow diagram of the trial is shown in **Figure 2**.

#### 2.5.3 Emergency treatment

Participants who are unable to tolerate their LARS symptoms or are dissatisfied with the treatment effects will, at their request, have the option to receive loperamide Hydrochloride Capsules (Imodium, Xi’an Janssen Pharmaceutical Co., LTD) (17), the guideline-recommended first-line medication for LARS. Detailed recording of medication dosages and treatment duration will be maintained in the case report forms (CRFs).

#### 2.5.4 Prohibited concomitant medications/treatments

During the trial, with the exception of medications/treatments permitted by the protocol, the following interventions that may interfere with study assessments are prohibited, including but not limited to: (1) anti−diarrheal agents, such as ramosetron, ondansetron, and compound diphenoxylate tablets; (2) TCM therapies for LARS, including oral administration of other Chinese herbal medicines, acupuncture, acupoint application, moxibustion, acupoint catgut embedding, among others; and (3) other Western medical interventions, such as neuromodulation, pelvic floor rehabilitation therapy, and transanal irrigation.

### 2.6 Randomization, allocation concealment, and blinding

The randomization schedule for both participants and investigational products was generated using SAS software (version 9.4). Subject randomization and drug allocation will be implemented through the Central Randomization System V5.0 (provided by Beijing Medfuture Technology Co., Ltd.). Eligible patients will be randomized in a 1:1 ratio to receive either the BL-1 group or the placebo group. Simultaneously, the dosage form, properties, odor, appearance, packaging, labeling, and identification of the placebo will be matched with those of the treatment group, and clinical pharmacists prepared the corresponding medications using random numbers to implement allocation concealment for bias minimization (18). All participants and investigating clinicians will remain masked to treatment assignment throughout the trial duration until the final database lock. Designated personnel from the Evidence Based Medicine Centre of West China Hospital will have exclusive access to the randomization list and are mandated to maintain confidentiality until trial completion and full data collection, unless unblinding is necessitated by serious adverse events.

Unblinding of a participant’s assigned intervention will constitute grounds for withdrawal from the study, and the case will be documented accordingly. If 20% or more of the required sample size has their assigned intervention disclosed, the trial can be considered to have failed in maintaining blinding for participants (19). To uphold objectivity and minimize potential bias from investigators and participants, the statistical analysis for this trial will be performed by an independent third party under a blinded manner.

### 2.7 Outcomes

#### 2.7.1 Primary outcomes

The primary outcome is defined as the change in LARS score between baseline and week 4. The degree of LARS symptom severity will be measured using the LARS symptom severity score (LARSS) (20). The LARSS is assessed across five key domains: incontinence for flatus, incontinence for liquid stool, stool frequency, clustering of bowel movements, and urgency. The summation of individual item scores yields the total score, which is subsequently categorized according to predefined severity levels. The LARSS will be used to stratify disease severity into three levels: normal (score ≤ 20), mild (21 ≤ score ≤ 29), and severe (30 ≤ score ≤ 42). Treatment response will be evaluated based on changes in severity from original (baseline) level. Patients showing no improvement or any deterioration (e.g., from mild to severe) will be classified as non-responders, while those demonstrating improvement will be categorized as responders (e.g., from severe to mild, from severe to normal, and from mild to normal). The response rate is defined as the ratio of responding participants to the total number of randomized participants, multiplied by 100%.

#### 2.7.2 Secondary outcomes

Secondary endpoints will include the following: (a) changes in the LARSS from baseline to weeks 2 and 6; (b) the proportion of patients demonstrating improvement in bowel dysfunction across the three assessment time points (weeks 2, 4, and 6). Clinical transitions in LARS severity will be assessed using the LARSS, specifically documenting the number of patients transitioning from severe to mild, mild to none, and severe to none; (c) stool frequency, which will be assessed by recording the average number of spontaneous bowel movements per day or per week for the patients at weeks 0, 2, 4, and 6; (d) stool consistency, which will be assessed by recording the average daily or weekly stool consistency at weeks 0, 2, 4, and 6 (21); (e) anal function, which will be assessed using the Wexner anal incontinence scale score at weeks 0, 2, 4, and 6 (22); (f) QOL, which will be assessed at weeks 0, 4, and 6 using the cancer-specific European Organization for Research and Treatment of Cancer scale (EORTC QLQ-C30) (23); (g) functional status, which will be assessed using the Karnofsky Performance Status (KPS) score at weeks 0 and 4; and (h) safety, which will be evaluated as follows: 1) biochemical indices, including liver function (alanine aminotransferase and aspartate aminotransferase), kidney function (blood urea nitrogen and creatinine); 2) electrocardiographic assessment of cardiac electrical activity; 3) adverse events (AEs): any unfavorable symptoms (including rash, constipation) documented during treatment. AE incidence: (number of patients experiencing AEs/total number of patients) × 100%; 4) severe AEs.

### 2.8 Sample size

On the basis of preliminary results from the pilot trial, the BL-1 treatment group had a score reduction of 9.75 ± 2.30 points, whereas the placebo control group had a reduction of 8.55 ± 2.84 points. The overall mean change in the LARSS was assumed to be 9.75 for the BL-1 group and 8.55 for the placebo group. The variances of the two groups were homogeneous, with a standard deviation of 2.5. A superiority design was adopted, with a two-sided test level set at α = 0.05 and a test power of 1-β = 0.9. A sample size calculation was performed based on a balanced design (1:1 allocation), indicating that 93 participants are required per group. Adjusting for an anticipated withdrawal rate below 20% (24), the target enrollment was planned as 117 patients per group, but the intention−to−treat (ITT) and per−protocol set (PPS) analysis sets will be determined based on the final sample size and completion data at trial end. The data collected in this trial will inform future sample size calculations and provide a foundation for the design of subsequent large-scale clinical trials.

### 2.9 Data collection and management

Data will be collected using CRFs throughout both the treatment and follow-up phases of the study. Electronic data will be entered and managed by data administrators at the Beijing eMedyun Software Co., Ltd. (https://edc.emedyun.com/) using management software (e.g., EDC Software). To ensure data integrity and confidentiality, all personally identifiable information will be replaced with study codes. A formal database lock will be implemented by the data management team following study completion. A five-year retention period for all paper records will be maintained post-publication, after which they will be securely destroyed.

### 2.10 Trial quality control

Trial quality will be regulated as follows: (a) an independent BL-1 Data and Safety Monitoring Board (DSMB) will be constituted by West China Hospital of Sichuan University to monitor trial conduct and ensure quality throughout the study duration. The BL-1 DSMB will provide independent oversight of the trial’s progress. Its key responsibilities include: ensuring timely documentation and reporting of adverse events; safeguarding participant rights and welfare through verification of informed consent procedures; and critically reviewing all accumulated study data; (b) designated personnel from the Centre for Evidence-based Medicine at West China Hospital, Sichuan University, will provide auxiliary supervision. This includes implementing rigorous quality control measures to verify data accuracy and completeness, facilitating the systematic identification of discrepancies, omissions, or entries requiring clarification or correction.

### 2.11 Statistical analysis

Analyses will adhere to the ITT principle, including all randomized patients in their as-randomized groups, provided they received at least one dose of the study intervention (BL-1 or placebo) and completed a minimum of one post-baseline assessment. The PPS and ITT analyses for the primary outcomes will be performed concurrently to verify the consistency of the treatment effects. In cases of discordance between the ITT and PPS, the ITT results will be accorded primacy as the primary analysis for outcome interpretation. The robustness of the trial findings will be substantiated by concordance between the ITT and PPS. The ITT set will be employed for the analysis of baseline characteristics, while the safety set will serve for all safety evaluations. Missing data pertaining to primary outcome measures will be handled through multiple imputation techniques, implemented using R software (version 4.1.2). For remaining categorical variables, no imputation will be performed for missing data, and all available cases will be analyzed as observed.

Continuous variables such as LARSS, EORTC QLQ-C30, and KPS will be subjected to normality hypothesis tests. Continuous variables will be reported as mean (SD) or median (IQR), depending on distributional assumptions assessed through normality tests. Simple and multiple linear regression models will be employed to assess group differences between the BL-1 and placebo groups for both primary and secondary continuous outcomes. Two pre-specified adjusted analyses will be conditionally performed, contingent upon meeting prespecified conditions in the data. The first preadjusted analysis will incorporate *a priori* selected baseline variables, namely age, sex, smoking history, alcohol consumption status, radiotherapy history, and surgical characteristics. The second preadjusted model will further include concomitant treatment as an additional covariate, which is considered a prognostic factor unaffected by treatment assignment. Prespecified subgroup analyses will include the TNM staging of rectal cancer, baseline LARS severity, and three types of surgical resection: low, medium, and high. Categorical variables will be presented as frequencies and corresponding percentages. Primary and secondary categorical outcomes will be analyzed using chi-square tests, supplemented by logistic regression when conditions warrant its application. Adjusted analyses will be extended to categorical outcomes using the same predefined set of covariates as employed in the continuous outcome analyses.

The statistical software employed for data analysis will be IBM SPSS Statistics, Version 29.0. Estimates of intervention effects will be reported as point estimates accompanied by 95% confidence intervals, with a two-tailed *P* value threshold of < 0.05 defining statistical significance.

### 2.12 Ethics

The study protocol adheres to the ethical principles outlined in the Declaration of Helsinki and follows the Good Clinical Practice Guidelines. Any proposed amendments to the trial protocol shall be subject to discussion, consensus-reaching, and formal approval by the principal investigators. All protocol amendments will be concurrently submitted to the Medical Ethics Committee for re-approval and to the trial registry for official updates. The dissemination of trial findings will be achieved via publication in peer-reviewed scientific journals.

## 3 Discussion

LARS has been described as ‘disordered bowel function after rectal resection, leading to a detriment in QOL’ (5). Recently, a large international consensus trilingual Delphi process with patients as the major stakeholders defined LARS as having at least one of eight symptoms (variable, unpredictable bowel function; altered stool consistency; increased stool frequency; repeated painful stools; emptying difficulties; urgency; incontinence; soiling), resulting in at least one of eight consequences (toilet dependence; preoccupation with bowel function; dissatisfaction with bowels; strategies and compromises; mental and emotional wellbeing; social and daily activities; relationships and intimacy; roles, commitments and responsibilities) after anterior resection (5). Numerous techniques have been used to improve the symptoms of LARS, but a specific treatment for LARS is lacking: (1) First-line therapy for LARS includes dietary modifications (avoidance of wine, cold beverages and spicy or stimulating food and soluble fiber) (4). The therapeutic approach primarily involves agents that target colonic hypermotility. In clinical practice, antidiarrheal medications (e.g., loperamide, atropine) are utilized based on their therapeutic rationale to suppress colonic peristalsis and possibly augment anal sphincter function. As a therapeutic agent, the 5-HT3 antagonist ramosetron may alleviate a cluster of symptoms, including reduced bowel frequency, decreased urgency, improved fecal continence, and attenuated postprandial contractions in the neorectum (25). Ondansetron can improve symptoms and QOL in patients with LARS (26). (2) If LARS persists despite the administration of the above therapy for > 6 months, the second-line approach includes pelvic floor rehabilitation (Kegel exercise training and anal sphincter exercise training) with biofeedback training: pelvic floor rehabilitation has been reported to be effective in improving fecal incontinence, stool frequency, and QOL (27, 28); similarly, biofeedback therapy was associated with significant improvements in incontinence scale scores, number of daily bowel movements, and anorectal manometry data (maximum resting pressure, maximum squeeze pressure, and rectal capacity) (29). (3) If a patient fails first- and second-line therapy for major LARS after 1 year of treatment, sacral nerve stimulation is recommended (30): The mechanism of sacral nerve stimulation is thought to involve the afferent nerves from the anorectum and central levels, decreasing antegrade colonic motility and increasing retrograde activity (31, 32). (4) A systematic review indicates that there is no “magic bullet” for LARS (33); concurrently, a single “stepwise program” study reported that 77 per cent did not progress further than diet and medication (34). Patients with more persistent or intractable symptoms and/or impaired quality of life from major LARS that persists after 2 years of therapy may require a definitive stoma and typically, permanent colostomy (4, 7). Therefore, the current landscape calls for a paradigm shift in the follow-up management of colorectal cancer, and more health care professionals should focus on late-stage adverse effects and develop new treatment modalities or approaches.

Prompted by the limited treatment options in current guidelines and documented AEs of available therapies, our team undertook a comprehensive investigation into the pathogenesis of LARS through the lens of TCM, while also systematically reviewing the evidence for TCM and acupuncture efficacy and safety (data unpublished). The current evidence regarding LARS treatments is substantially constrained by methodological quality, necessitating rigorously designed investigations to validate TCM’s therapeutic role. Accordingly, we employed a randomized, double-blind, placebo-controlled design to assess the therapeutic potential and safety of the BL-1 prescription in patients with LARS. The key pathogenic mechanism of LARS is that the disease remains in Yangming, and dampness, phlegm and stasis are intertwined. Therefore, this study aims to assess the therapeutic application of BL-1 in managing LARS across a 4-week intervention period. The clinical effectiveness and safety of BL-1 for the treatment of LARS will be evaluated on the basis of the daily frequency of diarrhea, stool consistency, Wexner anal incontinence score, LARSS, LARS response rate, QOL, KPS score, and reduction in the need for acute treatment.

The following limitations should be considered when interpreting the results of this investigation. First, a notable constraint is the absence of validated objective measures for standardized assessment of LARS outcomes. Inter-individual variability and perceptual differences in symptom reporting may introduce substantial variance in documented drug efficacy profiles. Second, treatment dissatisfaction or intolerability of LARS symptoms may adversely affect patient adherence, thereby increasing the dropout rate. Third, as some participants may reside in other provinces, the quality control of the “direct drug delivery to participants” process requires further refinement. To enhance patients’ comprehensive trial awareness, our study staff received specialized training in establishing effective communication channels and building therapeutic rapport.
